# Inheritance of mitochondrial DNA in humans: implications for rare and common diseases

**DOI:** 10.1111/joim.13047

**Published:** 2020-03-18

**Authors:** W. Wei, P. F. Chinnery

**Affiliations:** ^1^ Department of Clinical Neurosciences School of Clinical Medicine University of Cambridge Cambridge UK; ^2^ Medical Research Council Mitochondrial Biology Unit School of Clinical Medicine University of Cambridge Cambridge UK

**Keywords:** human mitochondrial DNA, mitochondrial bottleneck, mitochondrial disorders, mitochondrial DNA mutation, mitochondrial inheritance

## Abstract

The first draft human mitochondrial DNA (mtDNA) sequence was published in 1981, paving the way for two decades of discovery linking mtDNA variation with human disease. Severe pathogenic mutations cause sporadic and inherited rare disorders that often involve the nervous system. However, some mutations cause mild organ‐specific phenotypes that have a reduced clinical penetrance, and polymorphic variation of mtDNA is associated with an altered risk of developing several late‐onset common human diseases including Parkinson’s disease. mtDNA mutations also accumulate during human life and are enriched in affected organs in a number of age‐related diseases. Thus, mtDNA contributes to a wide range of human pathologies. For many decades, it has generally been accepted that mtDNA is inherited exclusively down the maternal line in humans. Although recent evidence has challenged this dogma, whole‐genome sequencing has identified nuclear‐encoded mitochondrial sequences (NUMTs) that can give the false impression of paternally inherited mtDNA. This provides a more likely explanation for recent reports of ‘bi‐parental inheritance’, where the paternal alleles are actually transmitted through the nuclear genome. The presence of both mutated and wild‐type variant alleles within the same individual (heteroplasmy) and rapid shifts in allele frequency can lead to offspring with variable severity of disease. In addition, there is emerging evidence that selection can act for and against specific mtDNA variants within the developing germ line, and possibly within developing tissues. Thus, understanding how mtDNA is inherited has far‐reaching implications across medicine. There is emerging evidence that this highly dynamic system is amenable to therapeutic manipulation, raising the possibility that we can harness new understanding to prevent and treat rare and common human diseases where mtDNA mutations play a key role.

## Graphical abstract

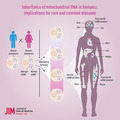

Content List ‐ Read more articles from the symposium: “Mitochondria in human disease”.

## Introduction: mitochondrial biogenesis – a tale of two genomes

Mitochondria are essential intra‐cellular organelles that are the primary source of energy in the form of adenosine triphosphate (ATP). Over 1000 different proteins are required to synthesize mitochondria and originate from two distinct genomes: nuclear DNA and mitochondrial DNA (mtDNA) [1. The nuclear genes are translated and transcribed in the cytosol, and proteins imported into the mitochondria combine with 13 essential peptides made within the mitochondrial matrix from mtDNA itself. There are multiple copies of mtDNA present within each mitochondrion. In addition to the polypeptide genes, 22 tRNA and 2 ribosomal RNA genes are also encoded, and are essential for intramitochondrial protein synthesis. Thus, the concerted action of these two genomes plays a fundamental role in cellular bioenergetics. If disrupted, this can cause cell dysfunction and human disease [1, 2, 3.

## mtDNA and human disease

**Fig. 1:**
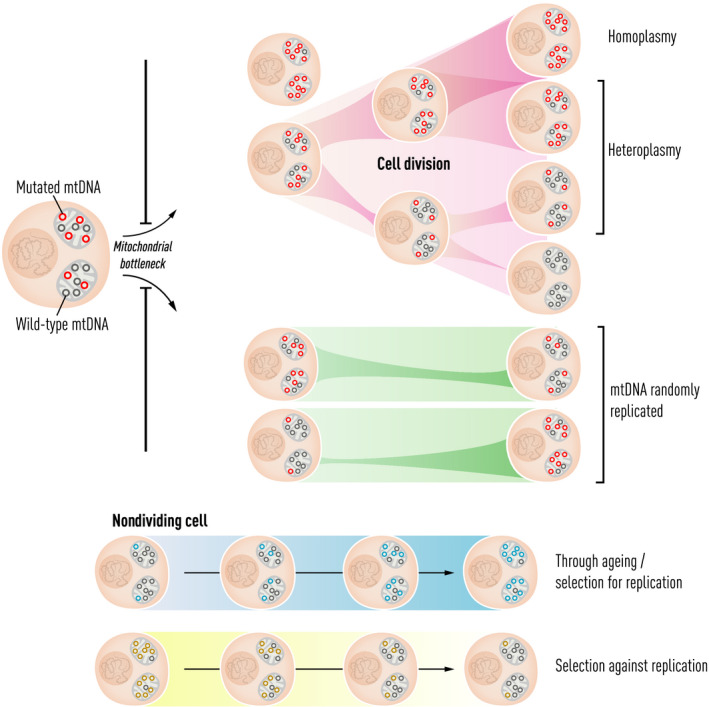
mtDNA genetic bottleneck and changes of heteroplasmy level throughout the lifetime. Each oocyte can inherit a different proportion of mutated mtDNA molecules from maternal mitochondria. When cells divide (shown in pink), heteroplasmy levels in each daughter cell can either increase, decrease or stay approximately the same. Once inherited, mtDNA mutations can continuously ‘clonally expand’ throughout life, even in nondiving cells (shown in green, blue and yellow). If one genotype is copied more frequently than another, it will change the overall proportion of different genotypes within the cell over time. The direction of this change can be influenced by selection for or against a particular mtDNA variant (shown in blue and yellow). When a mutated mtDNA molecule has a replicative advantage, the level will increase during life and possibly exceed the biochemical threshold, and thus contribute to the age‐related pathologies or the ageing process (shown in the blue box).

mtDNA mutations were first associated with disease in 1988 [4, 5. Patients with chronic progressive external ophthalmoplegia and the Kearns–Sayre syndrome have large‐scale deletions of mtDNA [5, 6, 7 (Table 1). The deletions were heteroplasmic, with individuals harbouring a mixture of mutated and wild‐type mtDNA. At around the same time, the m.3243A>G tRNA^Leu(UUR)^ gene mutation was described in Japanese families with mitochondrial encephalomyopathy with lactic acidosis and stroke‐like episodes (MELAS) [8, and the m.8344A>G tRNA^Lys^ mutation was described in families with myoclonic epilepsy with ragged‐red fibres (MERRF) [9, 10 (Table 1). Both mutations were also heteroplasmic, with family members harbouring a mixture of mutated and wild‐type mtDNA with varying proportions in different individuals. Heteroplasmic pathogenic mtDNA mutations characteristically display a threshold effect [11, where single cells must harbour a high proportion of mutated molecules before they affect oxidative phosphorylation in the production of ATP. From this earlier stage, it became clear that individuals inheriting a high proportion of mtDNA heteroplasmy were more likely to have a severe disease than individuals with low levels of mtDNA heteroplasmy (Fig. 1). Most heteroplasmic mtDNA mutations are inherited, but mtDNA deletions are rarely transmitted [12 for reasons that are not understood (Table 2).

**Table 1:** Clinical syndromes caused by mitochondrial DNA mutations

Clinical syndrome	Clinical features	Age of onset	Genetic basis
Chronic progressive external ophthalmoplegia (CPEO)	Ptosis, ophthalmoparesis. Proximal myopathy often present. Various other clinical features variably present	Any age of onset. Typically more severe phenotype with younger onset	mtDNA single deletions mtDNA point mutations (including m.3243A>G, m.8344A>G)
Kearns–Sayre syndrome (KSS)	PEO, ptosis, pigmentary retinopathy, cardiac conduction abnormality, ataxia, CSF elevated protein, diabetes mellitus, sensorineural hearing loss, myopathy	<20 years	mtDNA single deletions
Leber hereditary optic neuropathy (LHON)	Subacute painless bilateral visual failure Males:females approx. 4:1 Dystonia Cardiac pre‐excitation syndromes	Median age of onset 24 years	mtDNA point mutations (m.11778G>A, m.14484T>C, m.3460A>G and other rare point mutations in ~5%)
Mitochondrial encephalopathy, lactic acidosis, stroke‐like episodes (MELAS)	Stroke‐like episodes with encephalopathy, migraine, seizures. Variable presence of myopathy, cardiomyopathy, deafness, endocrinopathy, ataxia. A minority of patients have PEO.	Typically <40 years of age but childhood more common	mtDNA point mutations (m.3243A>G in 80%, m.3256C>T, m.3271T>C, m.4332G>A, m.13513G>A, m.13514A>G)
Myoclonus, epilepsy, and ragged‐red fibres (MERRF)	Stimulus‐sensitive myoclonus, generalized seizures, ataxia, cardiomyopathy. A minority of patients have PEO.	Teenage or early adult life	mtDNA point mutations (m.8344A>G most common; m.8356T>C, m.12147G>A)
Neurogenic weakness with ataxia and retinitis pigmentosa (NARP)	Ataxia, pigmentary retinopathy, weakness	Childhood or early adult life	*MTATP6* point mutation (usually at m.8993)

**Table 2:** Genetic counselling for mitochondrial DNA disorders

mtDNA mutation	Homoplasmy or heteroplasmy	Inheritance pattern	Recurrence risks
Large deletions	Heteroplasmic	Sporadic	<1/24 Transmission may be through an intermediate duplicated mtDNA molecule
Point mutation	Homoplasmic	Maternal	Variable reduced penetrance. Evidence that environmental exposures modulate penetrance. Male predominance in Leber hereditary optic neuropathy (LHON)
Point mutation	Heteroplasmic	Maternal	Variable penetrance correlates with the heteroplasmy level
Point mutation	Heteroplasmic	Sporadic	Tissue‐specific mutations typically affect skeletal muscle only, with low/no recurrence

**Fig. 2:**
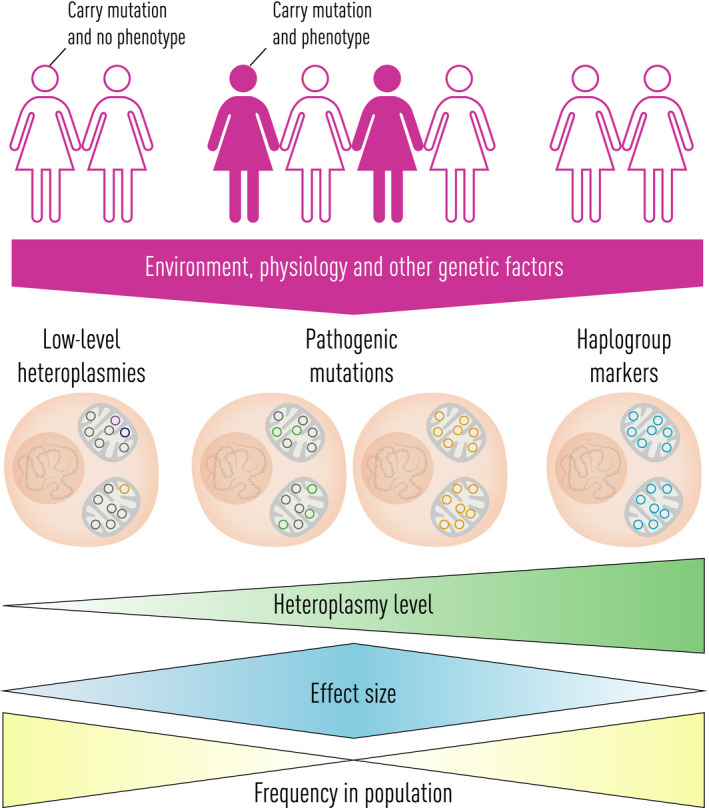
mtDNA variations in human diseases. Rare severe heteroplasmic mtDNA mutations usually have a strong effect size (middle left) (e.g. m. 3243A>G mutation in MELAS, see text). Conversely, some common mtDNA haplogroup‐associated variants have very weak effect size (right) (e.g. Parkinson’s disease). There are other variants have an intermediate effect size (middle right) (e.g. m. 11778A>G, m.3460A>G and m.14484T>C in LHON, see text).

In parallel, maternally transmitted mtDNA mutations were also described in Leber hereditary optic neuropathy (LHON), with the three most common affecting respiratory chain complex I (m.11778A>G in *MTND4*, m.14484T>C in *MTND6*, and m.3460A>G in *MTND1*) [4, 13, 14 (Table 1). Most families with LHON only transmit mutated mtDNA (homoplasmy), but the disorder has a markedly reduced penetrance which predominantly affects males. Thus, many people harbouring what is ostensibly a pathogenic mutation do not develop any illness, implicating additional environmental or genetic factors in the aetiology of LHON and related disorders. Other organ‐specific diseases caused by homoplasmic mtDNA mutations also have variable clinical penetrance (e.g. m.4300A>G. and maternally inherited cardiomyopathy [15). Additional genetic, physiological and environmental factors are thought to explain the reduced and variable penetrance in these disorders [16. Many LHON in families belongs to a specific mtDNA population haplogroup (European haplogroup J) [17, raising the possibility that the mtDNA genetic background modulates the clinical expression of the disorder [18 (Fig. 2).

Based on these findings, several investigators looked to see whether homoplasmic polymorphisms of mtDNA found in many healthy individuals could alter the risk of developing common complex human disorders. Although many of the early studies were under‐powered and led to inconsistent findings [19, several recent investigations have confirmed that common polymorphic variations of mitochondrial DNA, often clustered into ‘haplogroups’, alter our risk of developing disorders including Parkinson’s disease [20, type II diabetes [21, Alzheimer’s disease [22 and other late‐onset disorders [23.

Thus, mtDNA mutations and polymorphisms can contribute to a wide range of human pathologies. However, they do not always do this in isolation. In LHON, individuals carrying mtDNA mutations known to cause LHON are at increased risk of developing the disorder if they are exposed to cigarette smoking or heavy alcohol consumption [24. Likewise, individuals carrying the m.1555A>G variant are at increased risk of developing sensorineural deafness if exposed to aminoglycoside antibiotics [25, 26. In keeping with these findings, common mtDNA haplogroups also influence the risk of surviving severe infection and sepsis [27. Thus, there is emerging evidence of a complex interaction between mtDNA mutations and variants of differing severity, with the environment to cause both rare and common human diseases in different contexts.

In parallel to these discoveries, several laboratories have identified apparently acquired mtDNA mutations in tissues and organs affected by several late‐onset disorders including type II diabetes, idiopathic cardiomyopathy and neurodegenerative diseases such as Alzheimer’s disease [28, 29. A combination of both mtDNA point mutations and large‐scale mtDNA deletions can accumulate with a different mutation in each cell. These mutations ‘clonally expand’ throughout life, reaching higher levels and affecting cellular bioenergetics, and thus potentially contributing to the underlying pathology. Although observations made in human tissues only showed an association between the mtDNA mutations and disease, the subsequent generations of animal models established a causal link [30, 31, where clonally expanded mtDNA mutations in single cells contribute to age‐related pathologies and may even contribute to the ageing process itself (Fig. 1).

Until recently, mtDNA heteroplasmy was assumed to be very rare, and as a consequence, the acquisition of clonally expanded mtDNA mutations during human life was assumed to have been driven by a somatic mutation process. However, the recent development of deep re‐sequencing techniques has shown that mtDNA heteroplasmy is extremely common [32, 33, raising the possibility that some of the clonally expanded mutations observed in older individuals were actually inherited as low‐level heteroplasmies and present at birth, albeit at very low levels [34. Thus, both heteroplasmic and homoplasmic mtDNA mutations can be inherited and contribute to both severe highly penetrant primarily genetic rare diseases and common multifactorial disorders in combination with other mechanisms. Understanding how these mutations are inherited has therefore far‐reaching implications for medicine.

## Principles of mtDNA inheritance in humans

### Is there exclusive maternal inheritance?

**Fig. 3:**
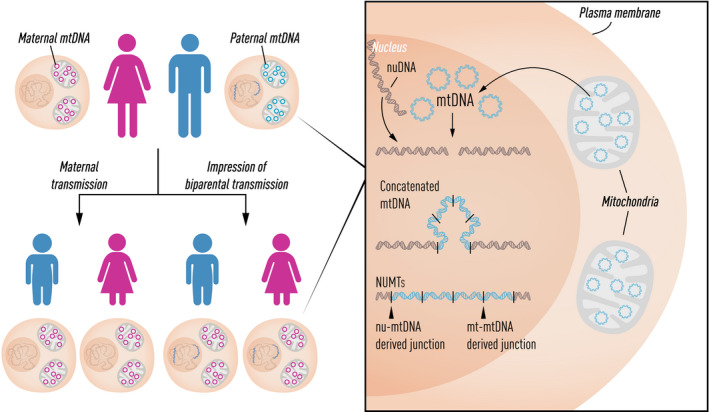
Illustration of nuclear‐encoded mitochondrial sequences (NUMTs) containing multiple concatenated copies of the mtDNA‐derived sequences inserted into the nuclear genome. A combination of NUMTs and mtDNA alleles can create the false impression of paternal inheritance of mtDNA.

Cytoplasmic genomes, which include mtDNA, are all almost always exclusively inherited from one parent. In some species, it is the male, but in most species, it is the female. There are unusual examples in nature where mtDNA can be inherited bi‐parentally, and intriguingly, this can change in response to environmental constraint. Paternal transmission has been documented in sheep *Ovis aries* [35 and the Great tit *Parus major* [36, but the ‘leakage’ of paternal mtDNA during transmission is seen in highly unusual situations, such as inter‐species breeding in mice [37, and *in vitro* embryo manipulation in cattle (*Bos taurus*) [38. In humans, there have been similar exceptions. The first description was in a patient with a mitochondrial disease caused by a two base pair deletion of mtDNA that was not detectable in either parent, but occurred on a paternal haplotype [39. Subsequent larger studies in other patients with mtDNA deletions failed to reproduce these findings [40, 41. However, the recent description of three families has rekindled the discussion. Luo *et al.* observed three multi‐generational pedigrees where a paternal haplotype was transmitted down several generations [42. Intriguingly, the paternal haplotype showed a high allele frequency (up to 40% of the alleles detected), with the allele frequency remaining stable down several generations. The authors presented these cases as examples of bi‐parental inheritance of mtDNA, but there has been considerable debate on this issue in the literature. Several alternative explanations have been proposed [43, 44, including the transmission of nuclear‐encoded mitochondrial sequences (NUMTs) [45 down the paternal line, creating the impression of heteroplasmy, when in actual fact the mixed haplotype is a combination of nuclear and mtDNA alleles (Fig. 3).

We recently looked for similar evidence of bi‐parental transmission of mtDNA in 11 035 trios [46]. We identified the signature in seven trios and showed that in these cases, the explanation was likely a paternally transmitted NUMT. The high allele frequency could be explained by the NUMT being complex, with multiple concatenated fragments of mtDNA (Fig. 3). Several authors [45 have argued this provides a more parsimonious explanation for the original findings of Luo *et al*. One could speculate that in some families, there is an inherited predisposition to paternal mtDNA transmission, perhaps through the genetic disruption of the normal mechanisms that have evolved to prevent bi‐parental transmission (e.g. by preventing the ubiquitin‐tagging of sperm mitochondria shortly after fertilization, and thus stopping the destruction of sperm mitochondria and mtDNA by autophagy/mitophagy). However, on theoretical grounds, some have argued it is a highly unlikely explanation [47.

Based on current evidence, it seems highly unlikely that paternal transmission occurs, and if it does, it must be exceptionally rare [48. Thus, any paternal leakage is unlikely to compromise the widespread use of mtDNA in anthropological and population genetic studies.

### Heteroplasmy and the genetic bottleneck

Initial observations in Holstein cows showed that a new mtDNA variant could be heteroplasmic in one cow, but rapidly become fixed down the maternal line within a few generations [49, 50. This led to the genetic bottleneck hypothesis, which predicted that only a small proportion of the mtDNA in a mother was used to repopulate the offspring of the next generation (Fig. 1). Mathematical analysis of heteroplasmy shifts observed across several vertebrate and invertebrate species supported the existence of an mtDNA genetic bottleneck, and experimental work in mice has cast light on the timing and underlying cellular mechanisms (comprehensively reviewed in Ref. [51).

Jack Jenuth and Eric Shoubridge’s observations in the mid‐1990s showed that the variance in heteroplasmy levels seen amongst siblings was determined during the development of the mother’s oocytes [52. This implied that the genetic bottleneck was occurring before the formation of oogonia, at least in large part. Analysing the mtDNA content of single cells, two laboratories subsequently showed a dramatic reduction in the mtDNA content in primordial germ cells just after their induction at day ~7.5 postconception in developing female mouse embryos [53, 54. Modelling studies showed that this reduction in mtDNA content could, on its own, make a substantial contribution to the variants seen in oogonia, perhaps accounting for up to ~70% of the variants in heteroplasmy levels seen in offspring [53. These findings confirm the presence of an mtDNA genetic bottleneck, but do not exclude the possibility that other mechanisms play a part, such as the compartmentalization of mtDNA molecules into individual nucleoids or mitochondria [55, 56, or additional mechanisms occurring after the formation of oogonia, perhaps involving the focal replication of mtDNA molecules [54.

A similar reduction in mtDNA content has been seen in several other vertebrate species including the sheep, zebrafish and salmon [57, 58, 59. Recently, it had been possible to isolate early primordial germ cells, from developing human embryos, and to generate primordial germ cell‐like cells from human embryonic stem cells *in vitro*. These findings confirm that a similar genetic bottleneck comes into play in the developing human germ line [60, contributing to the segregation of mtDNA variants in human pedigrees.

### Are all bottlenecks the same?

Empirical observations in human pedigrees transmitting heteroplasmic mtDNA mutations imply that there are genetic bottlenecks of different sizes, with some mutations showing faster segregation than others. This would explain why some mutations (e.g. m.8993T>G in *MT‐ATP6*) usually present in sporadic cases, having segregated from very low to high levels over one generation [61. On the other hand, some mutations (e.g. m.8344A>G in *MT‐TK*) show much more stable heteroplasmy levels with multiple generations being affected over time [10. A systematic evaluation of 577 mother–child pairs identified through the clinic from pedigrees transmitting pathogenic mtDNA mutations appears to support this conclusion [62, but these studies are very difficult to carry out because the pedigrees were identified through an affected individual. This introduces an ascertainment bias, where the proband in the most recent generation inevitably has a higher mutation load than individuals in previous generations where unaffected individuals did not reach medical attention. Although there are plausible ways that different mtDNA mutations could cause different genetic bottlenecks (e.g. by modulating the mtDNA copy number, as noted in the blood of individuals harbouring LHON mtDNA mutations [63), decisive evidence is lacking at present. It will be very interesting to see whether these findings can be reproduced in animal models transmitting mtDNA mutations affecting different genes.

### Selection for and against mtDNA variants

Studies of heteroplasmy transmission in human pedigrees imply that there may be selective forces acting during the transmission of mtDNA heteroplasmy. For example, a large population study in Finland raised the possibility that m.3243A>G levels progressively increase with subsequent generations [64. For the reasons explained above, it is very difficult to exclude the possibility that ascertainment bias explains the findings. Excluding the probands helps to mitigate against this problem, but does not completely remove the ascertainment bias. On the other hand, studying the transmission of mtDNA heteroplasmy in a number of mouse models supports the idea that there may be selection during transmission, particularly against high levels of mtDNA heteroplasmy. In extensive mouse pedigrees, Jim Stewart and colleagues showed clear evidence of selection against homoplasmic protein‐coding mtDNA variants [65. There is also evidence of selection against heteroplasmic mtDNA protein‐coding [66, 67 and tRNA gene mutations [68, 69 in mice.

Recent evidence supports the idea of selection both for and against variants in different genes in human pedigrees. Analysing 1526 mother–child pairs in the NIHR BioResource and UK 100 000 genomes project, Wei *et al.* studied the transmission of mtDNA heteroplasmy at levels well below the threshold typically thought to cause mtDNA disease [70. Analysis of these data was consistent with selection acting against the transmission of variants in the mtDNA ribosomal RNA genes, but there was also evidence of preferential transmission of mtDNA heteroplasmy in certain regions of the noncoding mtDNA D‐loop involved in the transcription and replication of mtDNA. Analysing heteroplasmy levels also suggested the signature of heteroplasmy acting against nonsynonymous mtDNA variants compared to synonymous mtDNA variants within the protein‐coding genes. Intriguingly, when mtDNA heteroplasmy was detected and transmitted, the variants were more likely to have been seen in population data gathered from across the globe. This implies that the selective forces acting during maternal transmission of mtDNA shape the genetic landscape of mtDNA in the human population [70. Studying individuals where the nuclear genome and the mtDNA have a different genetic ancestry (presumably because of an admixture event in the past), there is even the suggestion that the nuclear genome may be modulating this process, potentially under environmental constraint. These population‐based findings are supported by deep‐sequencing data from human embryos, where there is a signature of selection seen as germ cells proliferate and migrate from the hindgut region to the developing gonad between weeks 4 and 7 postconception [60. This coincides with a metabolic switch from dependence on glycolysis to oxidative phosphorylation, providing one potential way that negative selection might occur against variants that compromise ATP production. Alternatively, variants that promote mtDNA in their replication could be preferentially propagated during this time of intense DNA replication. Other mechanisms may be involved, including selective autophagy targeting organelles containing high levels of deleterious mtDNA variants, as shown recently in drosophila [71.

In conclusion, the dynamics of mtDNA heteroplasmy during germ cell specification appear to be shaped by selection, perhaps ensuring that the respiratory chain proteins encoded by the nuclear DNA and mtDNA remain compatible over the generations.

## Implications for Clinical Practice

Are these recent findings relevant for clinical practice (Table 2)? Based on current evidence, the paternal transmission of mtDNA appears to be exceptionally rare, if it occurs a tall. Thus, from a genetic counselling perspective, maternal transmission remains the established rule [72. For women harbouring pathogenic mtDNA mutations, homoplasmic mutations are transmitted to all of their offspring, and there are empiric recurrence risks for males and females based on extensive pedigree analyses for the more common mutations causing LHON. For other homoplasmic mutations, the recurrence risks are unclear at present, although accumulating data over the common years will likely address this issue. For women harbouring heteroplasmic mtDNA mutations, the recurrence risks for mtDNA deletions is low (~1 in 24) [12, but much higher for mtDNA point mutations. The presence of a genetic bottleneck creates considerable uncertainty, making it difficult to predict the outcome of pregnancy. There is growing experience using pre‐implantation and prenatal diagnostic techniques, but several laboratories have developed new approaches to prevent the transmission of mtDNA diseases by exchanging the nuclear genetic material, either through pro‐nuclear or chromosome spindle transfer [73, 74. These approaches have been used to prevent mtDNA disease in a Mexican child [75. However, there are theoretical concerns about the long‐term consequences, largely based on observations in in‐bred animal strains [76. In addition, several groups have seen the unexplained reversion of mtDNA heteroplasmy in human embryonic stem cells generated following mitochondrial transfer [73, 74. The reasons for this are not clear, and it certainly appears to occur more than one would expect simply by chance, raising the possibility that the original mutation is favoured by the nuclear genetic background. Further work is clearly required to clarify whether this is the case. In the longer term, observations in mice and drosophila raise the possibility that a more subtle nuclear–mitochondrial DNA mismatch might lead to late‐onset cardiometabolic traits [77 which in humans could take decades to emerge. Understanding the transmission of mtDNA heteroplasmy, the importance of the nuclear genetic background and the mechanisms of selection for or against specific variants will help inform our understanding of this process. It is also plausible that, by defining the mechanisms which favour transmission of one variant or another, it will be possible to influence heteroplasmy transmission using small molecules or other medical means, without recourse to mitochondrial transfer and the associated theoretical concerns. If this becomes a reality, then at some point in the future, it is conceivable that a similar approach would be used to prevent the inheritance of low‐level heteroplasmies in an attempt to reduce our lifetime risk of developing age‐related diseases, and possibly slow down the ageing process itself.

## Conclusion

For many years, the inheritance of mtDNA was thought to be simple and straightforward in humans. However, the recent discovery of near‐universal heteroplasmy, complexity introduced by the mtDNA bottleneck and evidence of selection for and against variants in particular regions of the mtDNA shows that the situation is far more complex than we previously thought. Given the emerging evidence implicating mtDNA mutations in the pathogenesis of common late‐onset diseases, and their possible contribution to the ageing process, a deeper understanding of these processes is key if we are to harness this knowledge and prevent and treat human disorders caused by mutations of mitochondrial DNA by manipulating their inheritance.

## Funding

PFC is a Wellcome Trust Principal Research Fellow (212219/Z/18/Z), and a UK NIHR Senior Investigator, who receives support from the Medical Research Council Mitochondrial Biology Unit (MC_UU_00015/9), the Evelyn Trust and the National Institute for Health Research (NIHR) Biomedical Research Centre based at Cambridge University Hospitals NHS Foundation Trust and the University of Cambridge. The views expressed are those of the author(s) and not necessarily those of the NHS, the NIHR or the Department of Health.

## Conflict of interest statement

No conflicts of interest were declared.
